# Supplementary material for: What polarizes citizens? An explorative analysis of 817 attitudinal items from a non-random online panel in Germany
Source: PLoS One. 2024 May 3;19(5):e0302446. doi: 10.1371/journal.pone.0302446 (PMC11068170; doi:10.1371/journal.pone.0302446)
Supplement: S1 Appendix — (DOCX) [file pone.0302446.s001.docx]

**Supporting information**

**Correlations**

S1 Fig shows that most covariates we coded are only weakly or moderately correlated. However, we see a relatively strong association between items we coded relating to minority issues and items including a notion of benefits (with a correlation of 0.52): items dealing with a minority group tend to measure a financial or non-financial benefit.


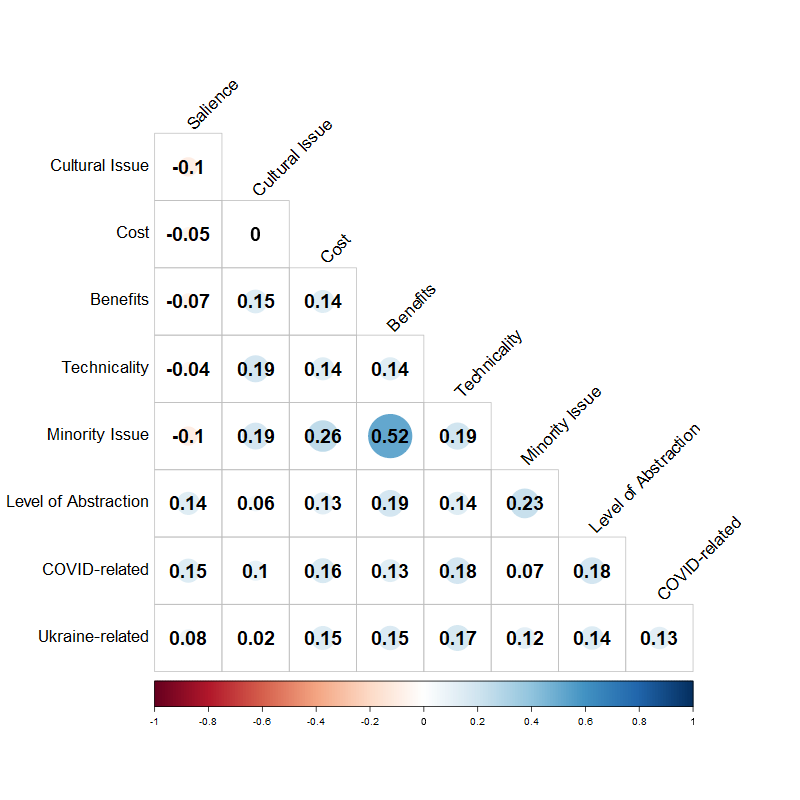


**S1 Fig. Correlation/Cramer’s V matrix.**

**Regressions**

S1 Table displays two regression models: In our first model, we see that the salience of an item and whether it is related to individual costs are significantly and negatively related to the van der Eijk index, which implies a higher level of polarization (and confirms our hypotheses 1 and 3). Items measuring cultural-related, minority-related issues or the level of abstraction do not affect polarization significantly. Therefore, we cannot confirm hypotheses 2, 4, and 5 in the multivariate model. By contrast, items with a medium level of technicality are significantly and negatively associated with the van der Eijk index, implying a higher level of polarization compared with items with a low level of technicality. We expected a negative association between cultural-related items and the van der Eijk index. Therefore, we reject our fourth hypothesis: items on gender, LGTBQI+, or immigrant and ethnic minority issues are associated with a higher level of *agreement* and not with a higher level of polarization, as hypothesized.

Turning now to the results of our second model, we can first observe that Ukraine- and COVID-related items are significantly associated with higher polarization among respondents. When including these two control variables in the model, we can observe that the coefficients for the other measurements remain stable, showing a robust association with opinion polarization.

**S1 Table. Multivariate Regression models of the Van der Eijk index.**

|  | Model 1 | Model 2 |
| --- | --- | --- |
| Salience (Log-Scale) | -0.036^**^ | -0.019^•^ |
|  | (0.011) | (0.011) |
|  |  |  |
| Cultural Issue | 0.022 | 0.022 |
|  | (0.039) | (0.038) |
|  |  |  |
| Cost | -0.104^***^ | -0.109^***^ |
|  | (0.026) | (0.026) |
|  |  |  |
| Benefits | -0.045 | -0.044 |
|  | (0.035) | (0.035) |
|  |  |  |
| Minority Issue | 0.005 | -0.003 |
|  | (0.034) | (0.033) |
|  |  |  |
| Level of Abstraction (Medium) | 0.031 | 0.025 |
|  | (0.022) | (0.022) |
|  |  |  |
| Level of Abstraction (High) | 0.051 | 0.024 |
|  | (0.037) | (0.036) |
|  |  |  |
| Level of Technicality (Medium) | -0.067^**^ | -0.071^**^ |
|  | (0.024) | (0.024) |
|  |  |  |
| Level of Technicality (High) | -0.031 | -0.062^•^ |
|  | (0.038) | (0.038) |
|  |  |  |
| Ukraine-related |  | -0.154^***^ |
|  |  | (0.031) |
|  |  |  |
| COVID-related |  | -0.168^***^ |
|  |  | (0.034) |
|  |  |  |
| Constant | 0.245^***^ | 0.296^***^ |
|  | (0.027) | (0.028) |
| Observations | 817 | 817 |
| R^2^ | 0.059 | 0.109 |
| F Statistic | 5.637^***^ (df = 9; 807) | 8.938^***^ (df = 11; 805) |

*Note*. •p<0.1, *p<0.05; **p<0.01; ***p<0.001

**Computing the Van der Eijk-Index**

The computation of the measure of agreement (van der Eijk 2001) can be summarised as follows:

- Firstly, decompose the empirical distribution into layers. Each layer consists of the same number of categories as the original distribution, and each category consists of either zero cases or exactly as many cases as the other categories. Therefore, these layers can also be described as semi-uniform components of the original distribution. They are then represented by binary terms, where 0s represent empty categories, and 1s represent non-empty ones. Please refer to van der Eijk (2001) for a graphical representation. An index $i=1, \ldots, K$ makes it possible to differentiate between the various layers.
- Calculate the measure of unimodality$U_{i}$ for any layer $i$ containing both 0s and 1s:

$$U_{i}= \frac{\left( K-2 \right)\times TU-\left( K-1 \right)\times TDU}{\left( K-2 \right)\times(TU+TDU)} ,$$

where K is the total number of categories in the rating scale, TU the number of triples of categories conforming to unimodality, and TDU the number of triples of categories deviating from an unimodal pattern. If layer $i$ consists of 1s only, then $TU=TDU=0$ and $U_{i}=1$.

- Agreement in layer $i$ is measured as follows:

$$A_{i}=U_{i}\times\left( 1-\frac{\left( S-1 \right)}{\left( K-1 \right)} \right) ,$$

where S is the number of non-empty categories in this layer, and K is the total number of categories in the rating scale.

- Finally, the layer-specific agreement measures are added up as follows:

$$A= \sum_{i} A_{i}\times w_{i} ,$$

where $w_{i}$ is layer $i$’s share of observations, serving as a weight.
