# Supplementary material for: What polarizes citizens? An explorative analysis of 817 attitudinal items from a non-random online panel in Germany
Source: PLoS One. 2024 May 3;19(5):e0302446. doi: 10.1371/journal.pone.0302446 (PMC11068170; doi:10.1371/journal.pone.0302446)
Supplement: Attachment — Submitted filename: Response_to_Reviewers_PONE-D-23-30566.pdf [file pone.0302446.s002.pdf]

Response Letter

What polarizes citizens? An explorative analysis of 817 attitudinal items from a non-random online panel in Germany

PONE-D-23-30566

This memo documents the changes we made to the paper in response to comments from the two reviewers. We want to thank the reviewers and the editor for the extremely helpful and constructive comments and the opportunity to send our revised paper for reconsideration. The comments were spot-on and helped us push the paper forward. The points raised by the reviewers are addressed one by one below (text in italics represents our answers to the concerns raised by the two referees):

Referee 1:

Referee 1's comments are very helpful in revising the paper. We hope we can satisfactorily address the general remarks and the comments concerning the theory and the empirical analysis.

A. General comments:

First, the reviewer encouraged us to revise our research question and to more systematically distinguish between aspects related to issues' substance and aspects related to survey item formulation. As she/they/he points out, we are not only testing whether the survey item formulation affects polarization but also whether the nature and substance of the issue that the items ask influences polarization. The reviewer states: "For instance, an issue's salience or whether it is a cultural issue do not depend on the survey item formulation. In contrast, whether it is abstract, or concrete can be a matter of item formulation."

Second, the reviewer raises the question of whether the study is specific to the German context or generalizable to other contexts.

Third, the reviewer asks what the advantage of including so many items is and if the analysis couldn't be done more efficiently.

1. Research Question

We followed referee's 1 suggestion and reframed our research questions. First, in the introduction, on p. 4, we now clarify our research questions: "We investigate the role of item characteristics in explaining the modality of the item response distribution. We take two characteristics into account: (a) the item's issue (e.g., the nature or substance of an item), and (b) the item's formulation."

Second, we reordered our hypothesis to match the separate research questions. Additionally, in the introduction, we altered a paragraph to read: "By drawing on the literature in psychology, sociology, and political sciences, we derive hypotheses on six item characteristics that we expect to influence distributive opinion polarization. The first two relate to the item's issue, the last four to the way items are formulated: (i) the extent to which an item issue was salient in the news media before the survey data collection, (ii) the extent to which an item tackles a cultural issue, (iii) the extent to which an item involves individual costs or benefits, (iv) the extent to which an item targets a minorty group, (v) the level of abstraction of an item, and (vi) the level of technicality of an item." Note that we also added a sixth hypothesis (see comments below in the section "F. On abstract vs. concrete formulation").

Third, we clarify again on p. 5 that we distinguish two sets of hypotheses: "While survey measurement research provides many general recommendations about survey item formulation (e.g., de Leeuw, Hox, and Dillman 2008; Schuman and Presser 1996), it does not specify the conditions under which a survey item is particularly likely to polarize respondents. We, therefore, draw on the literature in psychology, sociology, and political sciences to define two hypotheses on item issue characteristics and four hypotheses on item formulation characteristics and their role in distributive opinion polarization. In this section, we present our hypotheses on these item characteristics."

Lastly, we changed the abstract accordingly: "Various studies point to the lack of evidence of distributive opinion polarization in Europe. As most studies analyse the same item batteries from international social surveys, this lack of polarization might be due to an item's issue (e.g., the nature or substance of an item) or item formulation characteristics used to measure polarization. Based on a unique sample of 817 political attitudinal items asked in 2022 by respondents of a non-random online panel in Germany, we empirically assess the item characteristics most likely to lead to distributive opinion polarization – measured with the Van der Eijk agreement index. Our results show that only 20% of the items in our sample have some – at most moderate – level of opinion polarization. Moreover, an item's salience in the news media before the survey data collection, whether an item measures attitudes toward individual financial and non-financial costs, and the implicit level of knowledge required to answer an item (level of technicality) are significantly associated with higher opinion polarization. By contrast, items measuring a cultural issue (such as issues on gender, LGTBQI+, and ethnic minorities) and items with a high level of abstraction are significantly associated with a lower level of polarization. Our study highlights the importance of reflecting on the potential influence of an item's issue and item formulation characteristics on the empirical assessment of distributive opinion polarization."

2. Generalizability

The reviewer raises an important point regarding the generalizability of our study: Among the 817 items in our sample, only some of them are particular to the German

context (e.g., the implementation of a speed limit on highways). Most of the other items are relevant to many other contexts. Moreover, we controlled in follow-up analyses for the two dominant issues covered by the sampled items (COVID pandemic and war in Ukraine) and could find similar results than the ones presented in the paper. We, therefore, are confident that our main findings apply also to other contexts and for other periods. We added a paragraph in the conclusion to clarify how our results may also hold in other contexts. The paragraph (p. 26) reads: "While our study focused on survey items collected in a German non-probability online panel, we believe these findings will likely be generalizable to other contexts and periods. Indeed, while some items of our sample are particular to the German case (e.g., implementation of a speed limit on highways), the content of most of the 817 survey items is relevant to other countries and are likely to have been asked in a similar formulation in surveys in other countries. Moreover, the period in which the coded items were asked is characterized by two main issues (COVID pandemic and the war in Ukraine). Further robustness analyses show that our main findings remain stable even when controlling for these two major issues. Therefore, we are confident that our results will likely apply to other contexts and periods."

3. Inclusion of many items

The reviewer is totally right in pointing out that the inclusion of all the items comes at the cost of efficiency. However, we are convinced that from a substantial and methodological standpoint, the inclusion of a broad array of items provides a more comprehensive and detailed understanding of opinion polarization. We opted for the inclusion of all 817 items to allow for a more nuanced understanding of the phenomena. In our study, for instance, we find that only a minority (about 20%) of items tend to polarize public opinion. This result is significant as it challenges the general assumption of widespread opinion polarization and illustrates the importance of the analysis of an extensive sample.

B. On polarization

Reviewer 1 states that we "define polarization as a bimodal distribution. However, there are other definitions/ aspects of definitions of polarization that the authors do not engage with (e.g., see Traber et al. 2022). What about other aspects of polarization such as sorting or whether there is some kind of group identity? It would be great to see some engagement with this literature and justification of the chosen definition and potential limitations of this definition."

We thank reviewer 1 for highlighting this missing argument in our paper. We revised the introduction and the conclusion, mentioning the restriction of our study to distributive opinion polarization, which can be considered the most straightforward and least complex form of polarization. In the introduction (p.4.), we added the following clarification: "Arguably, we focus in this study on the simplest form of opinion polarization measured with the modality of an item's distribution among the

overall sample of survey respondents, leaving aside more complex forms of polarization, such as group-based polarization (Traber, Stoetzer, and Burri 2022) or affective polarization (Wagner 2021). This focus on a single and simple form of opinion polarization is necessary to launch a scientific debate targeting the role of item formulation and the issue of items in opinion polarization." In the conclusion, on p. 27, we added the sentence: "A further promising research avenue would be to assess the role of item formulation and item issue domains on other, more complex forms of polarization, such as group-based polarization (Traber, Stoetzer, and Burri 2022) or affective polarization (Wagner 2021)."

Further, reviewer 1 rightly challenges us on the account that opinion polarization is generally problematic. She/they/he writes: "Is opinion polarization always considered problematic, or can it be an indicator of "healthy" pluralism, too? Does this perhaps depend on the issue, e.g., when an opposition to an issue is problematic for democratic principles? Sometimes, general (dis)agreement with an issue could also be problematic from a democratic perspective, couldn't it?"

To account for this comment, we rephrased the first sentence of the introduction (p. 3): "Opinion polarization has become a prevalent research and mediatic topic in Western Europe over recent years, mainly due to its scientific and societal relevance. Indeed, while multiple and cross-cutting opinion polarization might be conducive to social order in pluralist societies, opinion polarization combined with issue alignment can lead to political conflicts and thus threaten social cohesion and social order (DellaPosta and Macy 2015; DiMaggio, Evans, and Bryson 1996)." However, we refrained from discussing the normative debate on opinion polarization at length, as it would go beyond the purpose of our empirical study focusing on item formulation and item issues.

C. On issue salience

Concerning issue salience, reviewer 1 raises two points: First, he writes: "The authors define issue salience as the coverage the news media affords a given issue. However, I would think that media coverage is a proxy/ measurement of issue salience rather than the definition of it. I would like to see an actual definition of what the authors mean by issue salience, and how media coverage captures it. Alternatively, the authors could specify that they only refer to salience in the media, rather than salience in general (see Wojcieszak et al., 2018)."

Second, the reviewer remarks: "As regards the effects of issue salience, I don't think that 'echo chambers' are the only possible mechanism at play in explaining the role of issue salience in polarization. For instance, issue salience leads to more availability of information and exposure to information, which increases the likelihood of people taking more determined positions on the issue. The issue salience literature (see Dennison 2019) can help with elaborating on such mechanisms."

We thank both reviewers for highlighting the limitations of the echo chamber theory for deriving our hypothesis on the role of media issue salience and distributive opinion polarization. In particular, we thank reviewer 1 for suggesting the work by Wojcieszak et al. (2018), which was indeed very relevant to us for rewriting the theoretical section for this hypothesis on media issue salience. We used a similar theoretical framework as the one proposed by Wojcieszak et al. (2018) to explain why mere exposure to issues through media is likely to lead to more radical opinion on an issue and, thus, to more distributive opinion polarization – based on the psychological theory of directly motived reasoning (Flynn, Nyhan, and Reifler 2017). Based on the reviewer's suggestions, we rewrote the section on issue salience on pp. 5–6: "Our first hypothesis on item issue characteristics refers to the role of media issue salience in distributive opinion polarization. From the psychological theory of directly motivated reasoning (Flynn, Nyhan, and Reifler 2017), we can hypothesize that mere exposure to information on an issue through the media is likely to lead to distributive opinion polarization. Directly motivated reasoning refers to the (unconscious) strategy of people to seek out information that reinforces their preferences (i.e., confirmation bias), denigrate attitudinal incongruent arguments (i.e., disconfirmation bias), and evaluate information supporting their prior attitudes as stronger and more compelling than counter attitudinal information (i.e., prior attitude effect) (Taber and Lodge 2006, 757). Directional motivational reasoning implies that processing additional information on an issue is likely to sharpen citizens´ prior beliefs and attitudes on the particular issue, which in turn increases attitudinal polarization (Taber and Lodge 2006). Empirical studies have indeed shown that directly motivated reasoning leads citizens to endorse stronger opinions (i.e., be more polarized) on an issue after having been exposed to new information on this issue. This effect appears in particular among those who have strong prior opinion on the respective issue and those who are more politically knowledgeable, as the former have affective links to the issue and the latter possess more ammunition to counter information disconfirming their prior beliefs (Taber and Lodge 2006; Wojcieszak, Azrout, and De Vreese 2018).

The (unconscious) activation of directly motivated reasoning is independent of the content of the information to which one is exposed (i.e., whether the information content confirms or disconfirms prior beliefs) (Taber and Lodge 2006). Thus, the mere exposure to media news on an issue is likely to induce directly motivated reasoning among citizens (in particular, those with strong prior beliefs and those more politically knowledgeable) who would then hold more polarized opinions on the respective issue. Indeed, Wojcieszak et al. (2018) showed that citizens in the Netherlands who were both fervent supporters and opponents of the EU held more polarized opinions after being exposed to media news about the EU. Therefore, we expect items with a high issue salience in the media to be more polarizing. By media issue salience, we refer to the relative coverage the news media allocates to a given issue (Epstein and Segal 2000)."

Third, the reviewer asked that Table 3 show the minimum and maximum values of the salience variable.

We revised Table 4 (formally Table 3) following the reviewer's suggestion (see pp. 21-22).

D. On loss aversion

Referee 1 raised to points concerning our cost/benefit variables. First: "I wonder whether the authors could make use of the literature on material and symbolic threats with regard to theorizing the effects of (perceived) losses/ costs." Second: "Further, I wonder about the context- and perception-specific nature of losses and benefits. For instance, the example mentioned by the authors on the highway speed limit entails the cost that people aren't allowed to speed on the highway, but it also entails a gain in road traffic safety. Similarly, the authors assess that lifting Covid test obligations is a benefit, however, this comes at the cost of a greater health risk. Whether an item is seen as entailing a cost or a benefit seems to depend a lot on individual perception. Such classification may thus be more ambiguous than proposed by the authors. I think it is safe to associate a cost with items specifically asking respondents about their willingness to pay for something, and to associate a benefit with tax incentives. However, I don't think that other items are easily classified as entailing a cost or benefit. Therefore, I do not trust the current coding of the measurement and I would recommend the authors to apply a stricter definition of cost and benefit in more strictly financial terms."

We considered the suggestion to incorporate the literature on material and symbolic threats but refrained from doing so. Our decision to not explicitly use this framework was based on the specific focus and methodology of our study. We concentrated on a more direct assessment of perceived costs and benefits as they relate to specific policy items. Our approach was to examine these perceptions through a more pragmatic lens, primarily considering immediate, tangible impacts.

As reviewer 2 raised a very similar point on the coding of our variable, we answered this point below on pp. 10-11 of this response letter.

E. On minorities

The reviewer asks: "Which groups do the authors consider as minorities? E.g. are women considered as a minority? Some would say that they are because of their discrimination, others would say that they aren't, because they are a large group in society. Similarly, not everyone would consider old people as a population group that is typically considered part of identity politics."

As we stated on p. 16, we understand minorities broadly: whenever an issue targets a clearly defined group, not the whole population, the variable minority is coded as 1. These two items are exemplary for the variable: "Should gay couples have the same adoption rights as heterosexual couples?"; and "Would you support a general Corona

vaccination requirement starting at age 60?" In this sense, both women and the elderly are considered as a minority in our analysis.

Further, a more restrictive definition of minorities encompasses too few cases, rendering it impractical. This results in the minority category being insufficiently representative for analytical purposes. Consequently, a significantly larger dataset is required to effectively assess a more specific definition of minorities.

F. On abstract vs. concrete formulation

Reviewer 1 raises an important point concerning our notion of an item being abstractly formulated: "How do the authors deal with the fact that some concrete items require a lot of specific knowledge to make an assessment? For instance, the question "Should the federal government recognize Jerusalem as Israel's capital?" requires knowledge about the implications of such a recognition. Similarly, the question "How do you currently rate the work of Federal Minister of Labor Hubertus Heil on a scale of 1 (very good) to 6 (insufficient)?" requires knowledge about the work of Hubertus Heil. What does it mean to include items of which respondents are very unlikely to give informed answers, and for which a good level of knowledge is necessary? Could a lack of polarization on such items indicate that people just don't know what to answer, rather than that they don't have strong opinions on them? I would suggest removing items that require a high level of specific knowledge."

We thank both reviewers for this brilliant suggestion! We honestly haven't considered an item's level of technicality as a further potential determinant of distributive opinion polarization. We followed reviewer 2's suggestion and included a sixth hypothesis on the item level of technicality. We use the theoretical framework provided by Carmines and Stimson (1980) on hard vs. easy issues and by Converse (1964) on citizens' level of political sophistication: The new section on pp. 11-13 reads:

"Our last hypothesis focuses on the role of item level of technicality in distributive polarization. For this, we draw on both the work of Carmines and Stimson (1980) on the distinction between easy and hard political issues and on the work of Converse (Converse 1964) on voters' level of political sophistication.

According to Carmines and Stimson (1980), the distinction between easy and hard political issues is essential for a better understanding of issue voting. Easy issues imply so-called gut responses, "Because gut responses require no conceptual sophistication, they should be distributed reasonably evenly in the voting population" (Carmines and Stimson 1980, 49). Thus, all citizens – regardless of their level of political interest, political knowledge, or level of education – possess the ability to express their own opinion when answering such easy issue items. By contrast, the discriminatory power of hard issue items is likely to be higher among citizens who are more politically interested, informed, and involved than among citizens less interested, informed, and involved. In other words, it will be mostly citizens with a high level of political sophistication and political interest who will be able to give a valid answer expressing

their own opinion on a hard issue. By contrast, citizens lacking political knowledge and involvement are more likely to give random answers to hard-issue items. Thus, item measurement error and consequently item variation are likely to be larger on hard issues than on easy ones. We would therefore expect more distributive polarization on easy issues than on hard issues as positions on easy issues are measured with more accuracy.

Converse (1964) comes to a similar conclusion in his seminal work on the nature of belief systems in the mass public when analyzing the implications of varying levels of political sophistication among voters. According to him, respondents with a lower level of political sophistication show political positions that are more random and less structured than respondents with a higher level of sophistication. He argues that a lack of political information and contextual grasp among citizens leads to the inability to relate one's ideology and own beliefs to a particular political issue. From an item perspective, items requiring a high amount of political and contextual information tend to show more variation and randomness in their answers than items not requiring such information (Converse 1964).

Carmines and Stimson (1980) conceptualized hard and easy issues by building on three complementary dimensions: level of technicality, measurement of policy ends and means, and length of the salience of an issue on the political agenda. We already drew hypotheses on two out of the three dimensions of easy and hard issues (i.e., hypothesis on level of abstraction -including the distinction between policy means and policy ends- and hypothesis on media issue salience). We, therefore, restrict this last hypothesis to the level of technicality of an issue, which enables us to construct a unidimensional indicator for measuring the easiness of an issue. Accordingly, technical issues require knowledge of important factual assumptions (Carmines and Stimson 1980). Hence, our last hypothesis is that the higher the level of technicality, the lower the level of polarization of an item:

H6: Items with a low level of technicality polarize more than items with a high level of technicality."

Additionally, we included this new variable in the operationalization section, the discussion of the results, and the conclusion.

The operationalization for this variable is stated as follows on p. 17: "We measured the level of technicality with three categories: Items in category 1 require from respondents a high level of political sophistication to give a valid answer. Examples for this category are: "How satisfied are you with the work of the Federal Minister of Construction, Klara Geywitz?" or "How do you evaluate the fact that the federal government wants to include stocks more in pension planning in the future". The second category comprises items with an intermediated required level of sophistication. Examples are "Should Turkey remain a NATO member?" or "How much confidence do you have in the German rule of law?". The third category is composed of items requiring the lowest level of technicality. Examples for the third category

include: "Would you describe yourself as a pacifist?" or "Does Islam belong to Germany?"

In the results we added the findings for our new variable. Note that given that we changed the sequence of our hypotheses, we altered the results section accordingly on pp. 18-23.

Further, we want to point out that the inclusion of the level of technicality as a control does not substantially alter the estimates for the other controls (see Table S1 in the appendix).

Referee 2:

We would like to thank Markus Wagner (reviewer 2) for his thoughtful comments on the paper. Addressing them strengthened it considerably. We hope that we could address Markus' concerns in the revised manuscript. In the following, we discuss his comments one by one:

A. Level of abstraction

The first comment reads: "When it comes to issues, I was also wondering whether other divisions may be useful. One key distinction often made in the literature is between easy and hard issues (Carmines & Stimson 1980). This is not quite the same as the level of abstraction. The argument is that some topics are 'easier' in that they require less complex answers – abortion or the death penalty are perhaps examples. The correct taxation policy is more of a hard issue. It surprised me that this common distinction was not considered or discussed. Similarly, I wondered about moralization as a related term that is used to distinguish different issues."

We also thank reviewer 2 for this brilliant suggestion, which was also raised by reviewer 1. We have already responded to this concern above in this response letter (see last comment, reviewer 1).

Regarding moralization as a term of distinction, we thoroughly considered adding a new variable capturing moralization as the process by which preferences are transformed into values, often making the issues more polarizing as they become tied to moral or ethical dimensions. Nonetheless, we opted not to code a new variable as, in our opinion, the items labeled as "cultural issue" – at least to a certain degree – capture this concept.

B. Salience

Reviewer 2 pointed out that "[T]he justification of the salience hypothesis is a bit odd. There is not a lot of evidence of echo chambers, at least online. Instead, in my view salience forces people to actually think about a topic and formulate their answer. Salience

also means that elites have provided useful (often partisan) cues. These are stronger reasons why salience matters."

We agree with reviewer 2 on the limitations of the echo chamber theory for deriving our hypothesis on the role of media issue salience and distributive opinion polarization. As this concern was also voiced by reviewer 1, we already responded to this comment above in this response letter (see section "C. On issue salience" above).

C. Financial / no-financial costs/benefits

The argument about non-financial costs and benefits is not clear. The example given makes it even less clear. How is a ban on imperial war flags a cost? It is not a cost for everyone. For many it would be a benefit! Maybe referencing clear policy proposals or policy change would be more useful.

Reviewer 2 raises a valid point: In our study, we classified non-financial costs and benefits based on their implications for individuals and society. We understand that certain measures, such as the ban on imperial war flags, can be perceived differently depending on one's perspective. We were not interested in the way an item might be interpreted, but rather on the way an item on a policy is formulated: is the item focusing on the costs or on the benefits of a policy? Most items on policies can be formulated by either using wordings tapping at the costs or the benefits (e.g.: "do you agree on the implementation of a new tax for X" uses the terms "implementation" and "tax", which refers to a cost. However, this policy could have been formulated in terms of benefits: "do you agree on increasing the budget for X by ….". We clarified this point by adding a paragraph on p. 16: "In our study, we operationalize "costs" and "benefits" by coding the exact wording used in the items. Obviously, most items formulating a financial or non-financial cost (for instance, the implementation of a new tax) could be formulated the other way around by stressing the financial or non-financial benefits of such a policy (for instance, increasing the financial budget that would result from implementing a new tax). Moreover, items formulated by highlighting a cost can be interpreted by respondents as introducing a benefit. However, and for the sake of consistency, we focused our coding exclusively on the wording used in the item formulation that tap at (financial and non-financial) costs or benefits."

Our decision to code all bans as costs was driven by a need for methodological consistency and by our focus on the formulation and wording of the items. In earlier versions of our paper, we explored a narrower conceptualization of costs and benefits. However, we found that a broader operationalization was necessary to maintain uniformity across various policy items (see also p. 16 of the manuscript).

To illustrate our methodology, consider other examples we coded: a proposal to increase taxes for environmental purposes was coded as a cost despite its potential long-term benefits to society. This consistent approach aids in avoiding subjective

biases in coding, especially given the complexity of disentangling the multifaceted impacts of each policy item.

This coding strategy does have implications for our study's findings. It suggests that our analysis might lean towards a more generalized view of policy impacts rather than a nuanced understanding of individual perceptions.

Further, we also differentiated between financial and non-financial costs or benefits in a previous version of our manuscript. Nonetheless, we opted for the broader operationalization as a further distinction comes as a cost of power in our analysis.

D. Minorities

Further, reviewer 2 raises the question about the relationship between the size and perception of a minority group and the extent to which it leads to polarization: "I am not sure minority targets should always lead to polarization, especially if the minority is small or strongly disliked."

Although our research suggests that minority targets may trigger polarization, we also note that this is not always the case. Indeed, the size of the minority group and how society generally views it are important considerations. For example, there may not necessarily be considerable polarization when there is a relatively small or strongly hated minority. The group's social influence or exposure might be too small in certain situations to generate widely held, polarising viewpoints.

But it's also critical to remember that, in some situations, even marginalized or despised minorities can serve as focal points for polarization. This may occur when these groups are brought up in public conversation, especially if it's done in a divisive or conspicuous way. In these circumstances, the minority group – regardless of its size or level of acceptance by the general public – may become a focal point or symbol for more significant social discussions, exacerbating polarization.

Nonetheless, in this case, we also tried to code the variable in a narrower manner, differentiating among different minority groups. However, we opted for this version of the variable as 817 items still are too little to further differentiate the minority that is targeted in the item.

E. Kappa

Reviewer 2 asked to "write a little more about common rules of thumb for Kappa agreement scores" and to specify what exactly the scores mean.

We specified on p. 18, based on Landis and Koch (1977a, 165), how to interpret the scores. The paragraph now reads: "To assess the intercoder reliability, we ran a Cohen's kappa test (Landis and Koch 1977b; 1977a). Table 1 shows the kappa values for our independent variables.

Table 1. Intercoder reliability.

Variable	*Kappa*
Culture	*0.76*
Benefits	*0.67*
Minority	*0.70*
Abstraction	*0.84*
Technicality	*0.65*

Following Landis and Koch's (Landis and Koch 1977a, 165) (p. 165) description of the relative strength of agreement associated with kappa (see Table 2), we obtained kappa values suggesting a substantial to almost perfect agreement.

Table 2. Kappa and strength of agreement.

Kappa	*Strength of agreement*
<0.00	*poor*
0.00 - 0.20	*slight*
0.21 – 0.40	*fair*
0.41 - 0.60	*moderate*
0.61- 0.80	*substantial*
0.81 – 1.00	*almost perfect*

F. Tables and Figures

Reviewer 2 pointed to two flaws in Table 1: First, we included the variable "majority" which we didn't consider in our analysis. Second, we didn't include the variable measuring whether the item is a cultural issue or not.

We corrected these two mistakes in Table 1: Majority is a variable we used in a previous version of the paper, and we mistakenly labeled the cultural dimension as "majority" in the table.

The last comment refers to Figure 3: "The distributions should be bar charts, as only 5 answer categories existed."

We thank reviewer 2 for his comments on Figure 3. However, it seems that there has been a misunderstanding about the purpose of this plot, as it shows the distribution of the polarization – a continuous variable – of all items within a topical category. A visualization using bar charts would be sensible for displaying the distribution of chosen answer options for a single item, but it is, as far as we can tell, not compatible with the purpose of Figure 3.

References

Carmines, Edward G., and James A. Stimson. 1980. 'The Two Faces of Issue Voting'. *The American Political Science Review* 74 (1): 78–91.

Converse, Philip E. 1964. 'The Nature of Belief Systems in Mass Publics'. In *Ideology and Discontent*, edited by David Apter, 206–61. New York: Free Press.

DellaPosta, Daniel, and Michael Macy. 2015. 'The Center Cannot Hold: Networks, Echo Chambers, and Polarization'. In *Order on the Edge of Chaos: Social Psychology and the Problem of Social Order*, edited by Edward J. Lawler, Shane R. Thye, and Jeongkoo Yoon, 86–104. Cambridge: Cambridge University Press.

DiMaggio, Paul, John Evans, and Bethany Bryson. 1996. 'Have American's Social Attitudes Become More Polarized?' *American Journal of Sociology* 102 (3): 690–755.

Epstein, Lee, and Jeffrey Segal. 2000. 'Measuring Issue Salience' 44: 66–83.

Flynn, D.J., Brendan Nyhan, and Jason Reifler. 2017. 'The Nature and Origins of Misperceptions: Understanding False and Unsupported Beliefs About Politics'. *Political Psychology* 38 (S1): 127–50.

Landis, J. R., and G. G. Koch. 1977a. 'The Measurement of Observer Agreement for Categorical Data'. *Biometrics* 33 (1): 159–74.

———. 1977b. 'An Application of Hierarchical Kappa-Type Statistics in the Assessment of Majority Agreement among Multiple Observers'. *Biometrics* 33 (2): 363–74.

Leeuw, Edith de, Joop Hox, and Don Dillman. 2008. *International Handbook of Survey Methodology*. London: Routledge.

Schuman, Howard, and Stanley Presser. 1996. *Questions and Answers in Attitude Surveys: Experiments on Question Form, Wording, and Context*. London: Sage.

Taber, Charles S., and Milton Lodge. 2006. 'Motivated Skepticism in the Evaluation of Political Beliefs'. *American Journal of Political Science* 50 (3): 755–69.

Traber, Denise, Lukas F. Stoetzer, and Tanja Burri. 2022. 'Group-Based Public Opinion Polarisation in Multi-Party Systems'. *West European Politica* 46 (4): 652–77.

Wagner, Markus. 2021. 'Affective Polarization in Multiparty Systems'. *Electoral Studies* 69: 102199.

Wojcieszak, Magdalena, Rachid Azrout, and Claes De Vreese. 2018. 'Waving the Red Cloth: Media Coverage of a Contentious Issue Triggers Polarization'. *Public Opinion Quarterly* 82 (1).
